# Characterization and Distribution of the *autB* Gene in *Neisseria meningitidis*

**DOI:** 10.3389/fcimb.2017.00436

**Published:** 2017-10-06

**Authors:** Aiyu Zhang, Pan Zhao, Bingqing Zhu, Fenglin Shi, Li Xu, Yuan Gao, Na Xie, Zhujun Shao

**Affiliations:** ^1^State Key Laboratory of Infectious Disease Prevention and Control, National Institute for Communicable Disease Control and Prevention, Chinese Center for Disease Control and Prevention, Beijing, China; ^2^Collaborative Innovation Center for Diagnosis and Treatment of Infectious Diseases, Hangzhou, China

**Keywords:** *autB*, *Neisseria meningitidis*, autotransporter, characteristic, distribution, DNA uptake sequence

## Abstract

We aimed to investigate and understand the characterization and distribution of the *autB* gene in *Neisseria meningitidis* in China. *autB* is flanked by two conservative genes, *smpB* and *glcD*, and it can be present in the majority of meningococcal isolates, but not in 053442 of clonal complex 4821 (CC4821) which contains a 968 bp sequence. In this study, we sequenced the intervenient region between *smpB* and *glcD* in 178 Chinese *N. meningitidis* strains isolated from both patients and carriers. There were 110 serogroupable strains, other 68 were non-groupable (NG). Ninety nine of the 178 strains were clustered into 13 CCs, the remaining 79 were unassigned (UA). CC4821 is one of the dominant CCs in China. Forty of the 42 CC4821 strains and 26 of the 79 UA strains were *autB*-null, while the remaining 12 CCs were *autB*-positive. According to the N-terminal sequence, most (97/112) of the *autB*-positive strains were clustered into AutB1 and the remaining 15 were AutB2. The *autB* gene and its flanking intergenic sequences was superseded by a perfectly conservative sequence of an identical 968 bp in all of the *autB*-null *N. meningitidis* strains which had no identity with the relatively conservative intergenic sequences that flanked the *autB* gene in *autB*-positive strains. There was a 10 bp DNA uptake sequence (DUS) at the beginning of the interval 968 bp sequence in the *autB*-null strains while there was a 9 bp *Haemophilus*-specific uptake sequence (hUS) at the beginning of the partial *holB* gene and at the end of the partial *tmk* gene in *autB*-positive strains, *holB* and *tmk* gene were flanking the *autB* gene in *Haemophilus*. In conclusion, not all pathogenic *N. meningitidis* strains especially CC4821 possess the *autB* gene in China and the corresponding spacer region of the *autB*-null strains was not homologous to that found in *autB*-positive strains. There's a hypothesis that the DUS and hUS are likely to play a key part in the mechanism of uptake or loss of the *autB* gene.

## Introduction

As a Gram-negative diplococcus, *Neisseria meningitidis* is an obligate human pathogen and it usually inhabits the nasopharynx, often asymptomatically. Nonetheless, this pathogen is also a common cause of meningitis and/or sepsis and it can generate large outbreaks. Autotransporters (ATs), which are synthesized by pathogenic Gram-negative bacteria, have been implicated in forming biofilms and may also correlate with virulence (Arenas et al., 2015; Arenas and Tommassen, 2017). To date, eight types of ATs have been studied in *N. meningitidis*. They are proteins that are usually composed of an N-terminal signal sequence, a secreted passenger domain and a C-terminal translocator domain (Grijpstra et al., 2013). The *iga* gene of the immunoglobulin A1 (IgA1) protease of *N. meningitidis* is single-copy and polymorphic, which suggests that it was attained through interstrain horizontal gene transfer (Lomholt et al., 1992). App is an acronym for adhesion and penetration protein which is homologous to the *Haemophilus* adherence and penetration protein (Hap). Similarly, the NalP is a Neisseria autotransporter lipoprotein which was historically named AspA (autotransported serine protease A) (St Geme et al., 1994; Turner et al., 2002; Serruto et al., 2003; van Ulsen and Tommassen, 2006). The functions of AusI (also known as MspA) have not been elucidated and its protein expression is influenced by NalP (van Ulsen et al., 2003, 2006; Turner et al., 2006). The former 4 ATs are monomeric and have protease activity whereas NadA and NhhA (Neisseria hia homolog A) are trimeric ATs that are related to host cell adhesion (Peak et al., 2000; Capecchi et al., 2005; Scarselli et al., 2006; Arenas et al., 2013). Other than the former four monomeric ATs, AutA is not secreted into the milieu of the bacteria and it also participates in autoaggregation (Arenas et al., 2015; Arenas and Tommassen, 2017). In contrast, the passenger domain of AutB is secreted but the AutB is still attached to the cell surface due to the translocator domain (Arenas et al., 2016).

A detailed study has elucidated the expression of the *autB* gene in *N. meningitidis*, whereby the mechanism of AutB appears to affect biofilm formation and epithelial transmigration (Arenas et al., 2016). A comprehensive analysis of AutB has been conducted and it complements the study of AutB, which has firmly assessed the ATs (Arenas et al., 2016). The distribution of the *autB* gene is well-known in the two pathogenic Neisseria spp. *N. meningitidis* and *N. gonorrhoeae*. There is no *autB* gene in *N. lactamica, N. flavescens, N. polysaccharea*, and *N. sicca* while some strains of *Haemophilus influenzae, H. haemolyticus, H. parainfluenzae*, and *H. aegyptius* are *autB* positive (Arenas et al., 2016). The number of AAGC tetranucleotide repeats (Rn) in the *autB* gene has previously been published and studies have considered whether the *autB* gene is in frame or out of frame depends on these repeats (Arenas et al., 2016). Although the *autB* gene is intact in most of the meningococcal strains (105/117), it is often out of phase, suggesting conservation but a very strong negative selection for AutB expression (Arenas et al., 2016). The amino acid sequence of AutB can be divided into three parts: the N-terminal part, the linker and the C-terminal translocator domain. The variability of AutB can be demonstrated by phylogenetic analysis of the N-terminal component and the linker domain and has been designated AutB1, AutB2, and AutB3, most of the variants are associated with AutB1 from *N. meningitidis* (Arenas et al., 2016). The phase variation was studied in patient strains of sequence type ST-32 clonal complex (CC32) (49 isolates) and CC213 (53 isolates) collected in Netherlands and 207 genome sequences of carriage strains in public data base, only one strain 2081107 (disease isolate) of CC32 have an intact *autB* gene and demonstrated in Western blotting, five strains of carrier isolates were also have an undisrupted *autB* gene, no obvious differences in *autB* expression between carriage and patient strains were demonstrated (Arenas et al., 2016).

It is noteworthy that the *autB* gene is omnipresent in the pathogenic *Neisseria* spp. except for 053442 (Arenas et al., 2016). 053442 is a serogroup C strain with the unique ST-4821, which was isolated from a patient in an epidemic of cerebrospinal meningitis in China (Shao et al., 2006; Peng et al., 2007). CC4821 was first isolated in China during 2003–2004 and then rapidly spread to two-thirds of the provinces within China (Shao and Zhu, 2016). Although CC4821 is one of the dominant hyperinvasive CCs in China, it has rarely been isolated in other countries (Zhou et al., 2012). The meningococcal A+C polysaccharide-based vaccine was introduced to the Expanded Program on Immunization (EPI) in 2005 and *N. meningitidis* invasive serogroup B soon increased after 2006 (Zhou et al., 2012). Meanwhile, the population structure of *N. meningitidis* invasive serogroup B were used to be mainly CC11 and CC41/44 and it evolved into CC4821 in China, CC4821 is one of the dominant hyperinvasive CCs in China now. It has been verified that there were capsular switching from *N. meningitidis* CC4821 serogroup C strains to serogroup B strains (Zhu et al., 2015).

The DNA uptake sequence (DUS) is a 10-bp (5′-GCCGTCTGAA-3′) non-palindromic sequence, which mediates the transformation of species-specific DNA (Goodman and Scocca, 1988; Rotman and Seifert, 2014). Twelve base pairs sequences (5′-ATGCCGTCTGAA-3′) have also been noted in pathogenic *Neisseria* (termed DUS12) (Ambur et al., 2007). It was first to show the presence of DUS bordering *autB* gene in 2001, in which *autB* was characterized as *lav* (Davis et al., 2001). Studies have also demonstrated that the DUS is sufficient for DNA uptake and transformation in previously non-transformable plasmids, while it can also competitively inhibit transformation when a DUS is already present (Goodman and Scocca, 1988; Elkins et al., 1991; Obergfell and Seifert, 2015). Therefore, the purpose of our work is to assess the distribution and characterization of the *autB* gene in *N. meningitidis* in China, especially for CC4821.

## Materials and methods

### Bacterial strains and DNA preparation

One hundred and seventy eight *N. meningitidis* strains obtained from 24 provinces in China from 1956 to 2015 were analyzed in our study. Sixty-seven of these strains were isolated from patients and the remaining 111 were isolated from healthy carriers. There were 27 strains isolated from 1956 to 2000, 31 strains from 2001 to 2005, 85 strains from 2006 to 2010, and 35 strains isolated from 2011 to 2015.

The bacteria were cultured on Blood Agar Media (Columbia) (PB0123A, Oxoid, China) at 37°C for 18–24 h in a 5% CO_2_ atmosphere. The genomic DNA of the strains was extracted using a Wizard Genomic DNA Purification Kit (A1125, Promega, Madison, USA) according to the protocol provided by the manufacturer.

### Serotyping, genotyping and MLST

Serotyping was performed on *N. meningitidis* strains by serum agglutination tests and rabbit antiserums against the 12 serogroups (BD, Sparks, USA; Remel, Kent, UK) were used (Xu et al., 2015). Genotyping was then implemented using PCR of the corresponding genes, the primers and amplification conditions have been described in former studies (Dolan-Livengood et al., 2003; Zhu et al., 2012; Xu et al., 2015). Multilocus sequence typing (MLST) was also performed on these strains according to the standard protocol (http://pubmlst.org/neisseria/) (Maiden et al., 1998; Maiden, 2006).

### Identification of the *autB* gene and bioinformatics analysis

As the genes flanking the *autB* gene are conserved in *N. meningitidis*, we gained the *autB* gene and its flanking sequences by PCR and sequencing of the PCR products. The dominant CCs of *N. meningitidis* in China are different to other countries, and the primers were redesigned according to the sequences of the *smpB* (NMB1526) and *glcD* (NMB1524) from the whole genome of *N. meningitidis* MC58 (Accession number NC_003112) (Tettelin et al., 2000; Shao and Zhu, 2016). The primer sequences are listed as follows: autB-F: 5′-GAAGGCTGGGAAGTCAAAG-3′ and autB-R: 5′-CGAAAACGACATCAACAGCAC-3′. To amplify the *autB* gene, TaKaRa LA Taq (RR02MA, Takara, Dalian, China) high fidelity polymerase was used and the annealing temperature was set to 57°C. The length of the products of MC58 ought to be 3,156 base pairs (bp) but it might fluctuate in other strains according to the number of AAGC repeats. In *autB*-null strains, the length of the products was considered to be ~1,539 bp according to the whole genome sequence of *N. meningitidis* 053442 (Accession number NC_010120). Both the PCR products with the positive and negative strains were sequenced by Beijing Tianyi Huiyuan Bioscience & Technology Inc, China. The alleles were aligned with the reference *autB* sequence of MC58 or the sequence of the spacer region in 053442 by MEGA (version 6.0) (http://www.megasoftware.net/). We then obtained the correct sequence of *autB* by excising the spare sequence (Tamura et al., 2013). The sequences of the spacer regions flanking the *autB* gene were obtained using the same method. The correct reading frame of *autB* was predicted by altering the number of AAGC repeats and removing the premature stop codons and frameshift mutations. Using the SignalP web tools (http://www.cbs.dtu.dk/services/SignalP/) to predict the cleavage site of the N-terminal signal sequence of AutB and neighbor-joining in MEGA, the phylogeny of the N-terminal domain of the passengers was analyzed (Arenas et al., 2016).

## Results

### Serotyping, genotyping, and MLST

Among the 178 *N. meningitidis* strains in this study, 6 serogroups (A, B, C, W, E, and X) and NG were identified. Twenty-one of the strains were serogroup A, 59 were serogroup B, 23 were serogroup C, 4 were serogroup W, 1 was serogroup E, 2 were serogroup X, and the remaining 68 were non-groupable (NG) strains. The genogroups of the 110 serogroupable strains were in accordance with their serogroups. The 68 NG strains were assigned to 6 genogroups and one group of capsule null locus (cnl) using PCR: genogroup B (22 isolates), C (18 isolates), E (7 isolates), W (5 isolates), Y (5 isolates), and X (3 isolates); eight were identified as being cnl.

These strains could also be clustered into 120 STs; 13 CCs and unassigned (UA) groups. Fifty-eight of these STs belonged to 13 CCs and another 62 STs were UA. Eight of the strains were CC1, 13 were CC5, 42 were CC4821, 8 were CC11, 8 were CC198, 79 were UA and the remaining 20 belonged to CC8 (1), CC32 (4), CC41/44 (4), CC92 (1), CC103 (1), CC174 (3), CC175 (5), and CC269 (1).

The characterization of the strains is listed in Table 1. Most of the strains are belonged to the dominant CCs in China, such as CC4821, CC5, CC1, CC11, CC198, and UA. All of the strains in CC1 and CC5 are serogroup A while other CCs distributed into many serogroups, especially the UA strains decentralized to serogroup B, C, E, X, and NG. The STs in the UA group are also flexible as there are 62 STs of the 79 UA strains.

**Table 1:** Characterization and distribution of the *autB* gene in *N. meningitidis* strains in China.

**Clonal complex**	**No.^a^**	**+/-^b^**	**Sequence type**	**Serogroup**	**AutB type**	**Rn^d^**
1	8	8/0	ST-3 (3^c^), other (5)	8 A	AutB1	3, 4
5	13	13/0	ST-7 (7), other (6)	13 A	AutB1	4
8	1	1/0	ST-5655 (1)	1 B	AutB1	5
11	8	8/0	ST-11 (5), ST-658 (2), ST-2724 (1)	4 W, 2 B, 2 C	AutB1	5, 8, 10, 11
32	4	4/0	ST-32 (1), other (3)	3 B, 1 C	AutB1	7, 10
41/44	4	4/0	ST-44 (1), ST-5635 (1), ST-8674 (1), ST-8918 (1, AutB2)	4 B	AutB1 (3), AutB2 (1)	4, 5, 7
92	1	1/0	ST-12774 (1)	1 NG	AutB1	4
103	1	1/0	ST-5944 (1)	1 X	AutB1	4
174	3	3/0	ST-6933 (3)	3 NG	AutB2	11
175	5	5/0	ST-175 (3), other (2)	1 C, 4 NG	AutB1	3, 5
198	8	8/0	ST-2146 (7), ST-8243 (1)	8 NG	AutB1	2
269	1	1/0	ST-5657 (1)	1 B	AutB1	5
4821	42	2/40	ST-4821 (15–, 1+), other (25–, 1+)	11 B (10–/1+), 14 C (13–/1+), 17 NG (–)	AutB1	4, 8
UA	79	53/26	ST-5586 (5), ST-5819 (3), ST-6934 (3), other (42); ST-5542 (4–), ST-5662 (4–), other (18–)	37 B (15–/22+), 5 C (2–/3+), 1 E, 1 X, 35 NG (9–/26+)	AutB1 (42), AutB2 (11)	3–8

a *Number of the strains*.

b *+, autB positive; –, autB negative*.

c *Numbers in the brackets are the quantity of strains in corresponding STs. In CCs include more than 4 STs, STs with less than 3 isolates were listed together as “other”*.

d *Rn, number of AAGC repeats in autB gene*.

### Distribution and characterization of the *autB* gene in China

One hundred and twelve of the 178 *N. meningitidis* strains were *autB*-positive and the other 66 were *autB*-null. The distribution of the *autB* gene is listed in Table 1 and detailed in Table S1.

The 21 strains of serogroup A were composed of eight strains of CC1 and 13 strains of CC5. All 21 strains were *autB*-positive.

Among the 59 strains of serogroup B, 22 strains belonged to 6 CCs, and 37 strains were UA. In the 11 strains of CC4821, only one strain of ST-4821 was *autB*-positive. This strain was 341215, isolated from the CSF of a patient in Anhui Province in 2012. The 11 strains of other five CCs were clustered into 11 different STs. They were all *autB*-positive. Among the 37 UA strains, 22 were *autB*-positive and distributed into 22 different STs. The remaining 15 strains of UA were *autB*-null strains and dispersed across 13 STs.

In serogroup C, there were 18 strains belonging to four CCs and five strains belonging to UA, all of the 23 strains were isolated after 2004. Fourteen strains belonged to CC4821. Only 440501, a ST-4831 strain, carried the *autB* gene. It was a clinical strain isolated from CSF in 2005. Another 13 strains without the *autB* gene were dispersed across 9 different STs. Three of the five UA strains were *autB*-positive, clustering into three STs. The remaining two were *autB*-null.

The four strains of serogroup W were ST-11, CC11, and *autB*-positive. The one strain of serogroup E was isolated from healthy carriers and clustered into ST-5586 (UA). The 2 serogroup X strains were all *autB*-positive.

All the 68 NG strains were isolated from healthy carriers. There were no genogroup A strains. The 22 genogroup B strains were isolated after 2005. Four of these were CC4821 and *autB*-null. The remaining 18 strains were UA, six of them were *autB*-null which belonged to six different STs. In genogroup C, all of the 11 CC4821 strains and three UA strains were *autB*-null, the remaining 4 UA strains were *autB*-positive. In the five genogroup W strains, 2 CC4821 strains were *autB*-null, the other 3 CC174 strains were all *autB*-positive. The 5 genogroup Y strains were *autB*-positive, 4 of them were CC175. The 7 genogroup E and 3 genogroup X strains were all UA and *autB*-positive. The 8 cnl strains were clustered into CC198, they were all *autB*-positive strains.

In conclusion, *autB* gene was only absent in strains of CC4821 (40 of 42) and UA (26 of 79), while an *autB* gene was detected in strains of the rest of CCs.

### Phase variation of the *autB* gene

The 112 *autB*-positive strains were analyzed. Without 9, the number of AAGC repeats in the *autB* gene were ranged from 2 to 11. Eight of the 112 strains were in frame according to the number of the AAGC repeats, seven of them with three tetranucleotide repeats and another one with six. In the Seven, the only one strain from patient was serogroup A, CC1 and there were no frameshifts, transposase or premature stop codons in its *autB* gene which indicated that AutB may synthesized in this strain. Other six strains were all isolated from carriers and they were all NG, 4 of them were genogroup Y of CC175 and another 2 were genogroup B of UA. Frameshift mutation was observed in all of the six strains. The other strain with six repeats was a NG strain belonged to genogroup X, it was isolated from carrier and clustered into UA. There were nothing wrong with the *autB* gene when turned to codons. In the 104 strains out of frame, the 8 cnl strains were all with 2 AAGC repeats and the 20 serogroup A strains were all with 4 repeats. The number of AAGC repeats maybe not strict to different serogroups.

### Structure and variability of AutB

**Figure 1:**
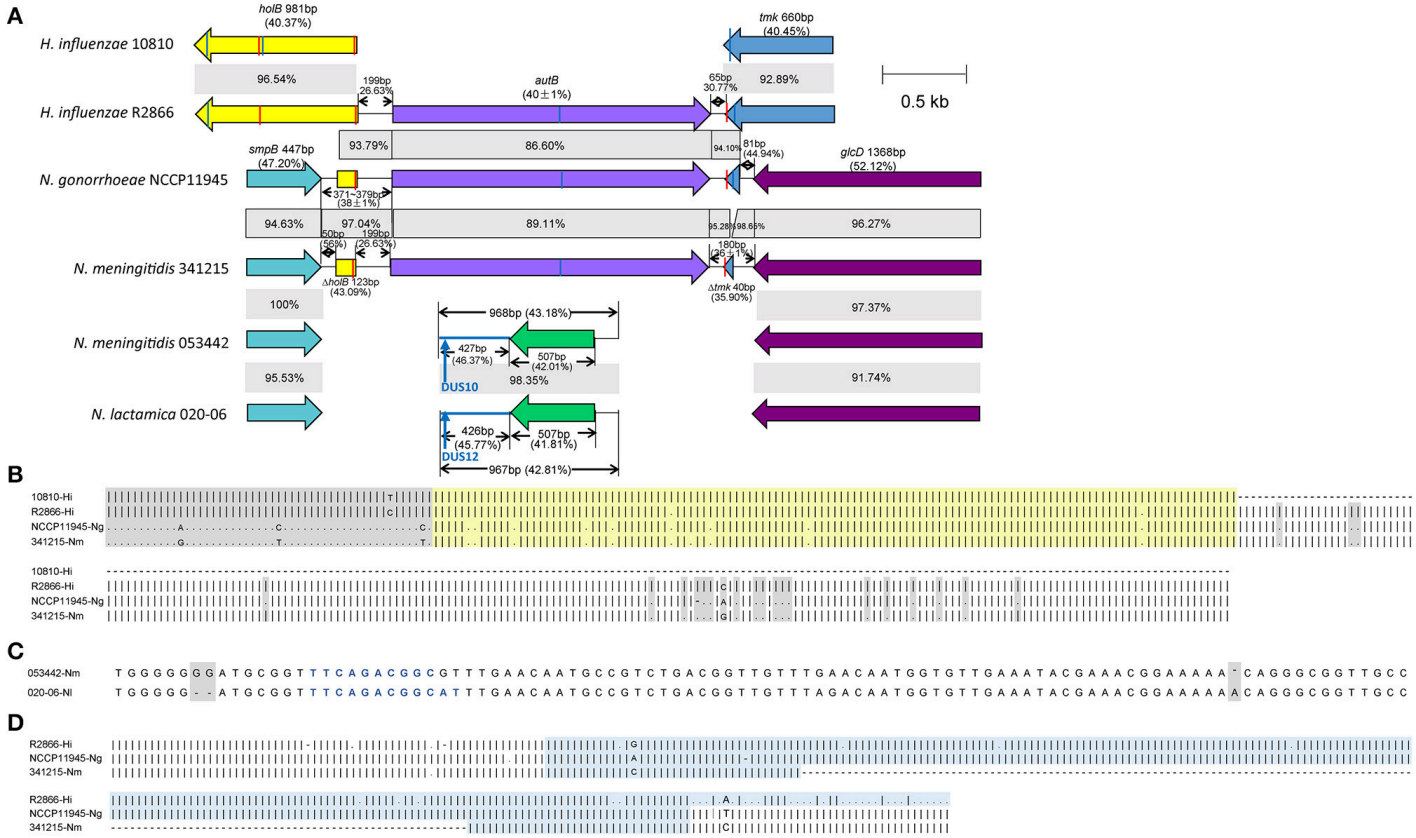
Comparison of the *autB* gene and its flanking genes. The *autB* gene and its flanking genes of *H. influenzae* 10810, *H. influenzae* R2866, *N. gonorrhoeae* NCCP11945, *N. meningitidis* 341215, *N. meningitidis* 053442, and *N. lactamica* 020-06 are compared. **(A)** The genes similar to each other are colored identically and the gray area represents the homology between the two sequences and the percentages in it represent the similarity between the two strains. The percentages in the brackets are the G+C content. The vertical bar in red represents the 9 bp hUS sequence of 5′-AAGTGCGGT-3′ and the vertical bar in blue represents the 9 bp hUS reverse complement to the former. In *N. meningitidis* 053442, the position of the blue arrow points from 16 to 25 of the 968 bp and is a 10 bp DUS sequence of 5′-TTCAGACGGC-3′ while in *N. lactamica* 020-06, it is a 12 bp DUS. **(B)** Alignment of the nucleotide sequence between *smpB* and *autB, H. influenzae* 10810 is *autB*-null, and there are ~123 bp in *autB*-positive strains of *N. gonorrhoeae* NCCP11945 and *N. meningitidis* 341215, which are homologous to *holB* in *H. influenzae*. This is highlighted in light yellow. The sequence in gray shadow demonstrates that nucleotides are different to each other. **(C)** Alignment of the nucleotide sequence between *smpB* and *glcD* in *autB*-null strains. There are only three nucleotide differences to each other which are in gray shadow and the nucleotides in blue are 10 bp DUS and 12 bp DUS, respectively. **(D)** Alignment of the nucleotide sequence between *autB* and *glcD* in *autB-*positive strains, shows there were 222 bp (highlighted in light blue) of the *tmk* gene in *N. gonorrhoeae* NCCP11945, which is homologous to that in *H. influenzae* R2866, while there were only 73 bp in *N. meningitidis* 341215.

The gene assignment of 106 of the 112 *autB*-positive strains was similar to *N. meningitidis* MC58, which has been studied in Arenas's paper. These strains might transcribe the *autB* gene after phase variation. In the other six strains, this was the same as the *N. gonorrhoeae* NCCP11945 (Figure 1). The arrangement for all 66 *autB*-null strains was identical to that observed in the *N. lactamica* 020-06, as previously described.

The analysis of the N-terminal part of the representative AutB passengers is displayed in Table 1 and Table S1. The phylogenetic trees of the N-terminal sequence of the *autB* gene in the 112 *autB*-positive strains and 10 strains from the NCBI database included *N. meningitidis* MC58, *H. influenzae* R2866, and F3047 were analyzed. There were also three branches of the N-terminal part of the AutB passenger and all 112 *autB*-positive strains in this study belonged to AutB1 and AutB2. However, except for the reference sequence of *H. influenzae* F3047, no AutB3 were found in our study. Most (97/112) of the strains with *autB* gene were clustered into AutB1. There were also many secondary branches of AutB1. Only three strains of CC174 (ST-6933), 1 strain of CC41/44 (ST-8918), and 11 strains of UA were clustered into AutB2.

There were 67 strains isolated from patients; 37 of these were clustered into AutB1, three were clustered into AutB2 and 27 were *autB*-null strains. In the 27 *autB*-null strains, only three UA strains were isolated in 1985 and 1988, while the other 17 strains (CC4821 and UA) were isolated after 2004. For the remaining 111 strains isolated from carriers, 60 of these were clustered into AutB1, 12 strains in AutB2 and 39 were *autB*-null strains. However, the correlation of AutB variability and different isolates was not clear.

### Characteristics of the spacer region flanking the *autB* gene

In *H. influenzae* R2866, the sequence of the spacer region between *holB* and *autB* was 199 bp with a G+C content of 26.63%, while between *autB* and *tmk* it was 65 bp with a G+C content of 30.77% (Figure 1). At the beginning of the *holB* gene, there was a 9 bp hUS within an extended 29 bp consensus sequence. At the end of the *tmk* gene, there was also a 9 bp hUS sequence. In *H. influenzae* 10810, a strain without the *autB* gene, the *holB* and *tmk* gene had a 4 bp overlap and there were also two hUSs on *holB* and *tmk*, respectively. In *N. meningitidis* MC58, which is identical to 341215 in the flanking sequences of *autB* gene, the sequence of the spacer region 1 between *smpB* and *autB* was 372 bp with a total G+C content of 36.29% while the spacer region two between *autB* and *glcD* was 180 bp with a total G+C content of 38.89% (Figure 1A). Both of the two spacer regions can be divided into three parts. In *H. influenzae* R2866, spacer region 1 consisted of 50 bp with a G+C content of 56.00%, 123 bp (43.90%) were homologous to *holB* gene and 199 bp (26.63%) were homologous to the sequence between *holB* and *autB* (Figure 1B). The length of spacer region 1 in the strains in China were 371, 372, or 379 bp (Figure 1A). The spacer region 2 was 180 bp and it consisted of 67 bp that was homologous to the sequence between *autB* and *tmk* with a G+C content of 32.84%; 73 bp was homologous to the *tmk* gene (42.47%); and 40 bp had a G+C content of 42.50% (Figure 1D). In *N. meningitidis* 053442 and another 66 *autB*-null strains in our study, the sequences between *smpB* and *glcD* were all 968 bp with a G+C content of 43.18% and were highly conserved with a 98% identity to *N. lactamica* 020-06 (Figure 1C). In the 968 bp sequence, there was a 10 bp DUS from 16 to 25. The 968 bp spacer region of *autB*-null strains was not homologous to the intergenic sequences flanking the *autB* gene in *autB*-positive strains. The *autB* gene and part of its flanking sequences in *N. meningitidis* in China are also homologous to the sequences in *H. influenzae*.

## Discussion

Microcolony formation is one of the strategies of *N. meningitidis* to elude the host immune response (Sim et al., 2000). The autotransporter AutB has been demonstrated to play a part in biofilm formation (Arenas et al., 2016). The *autB* gene was first described as an *orf2* downstream of *lsi1* (*rfaF*), a LPS biosynthesis related gene, and was then demonstrated to be horizontally-transferred from *H. influenzae* to *Neisseria* immediately before the bifurcation of *N. meningitidis* and *Neisseria gonorrhoeae*, where it was characterized as *lav* (Jennings et al., 1995; Davis et al., 2001). Subsequently, Arenas et al. have extended the analysis of the distribution of the *autB* gene and it is worth noting that in Arenas' study, *N. meningitidis* strain 053442 was the only clinical isolate without the *autB* gene. 053442 was a CC4821 strain and this clonal lineage has only been detected in China (Zhu et al., 2015). CC4821 has been a dominant lineage in China since 2003 (Zhou et al., 2012; Shao and Zhu, 2016). On account of this, we analyzed the isolates in China to further enrich the study of the *autB* gene.

We have not found any explicit relationship between the *autB* gene and the meningococcal serogroup. In a former study, all strains of the highly pathogenic CC213 and CC32 were *autB*-positive and these strains were clinical isolates; all 49 isolates of CC32 were clustered into AutB1 and 52 of the 53 isolates of CC213 were AutB1, while only one CC213 isolate harbored an AutB2 variant (Arenas et al., 2016). In our study, the 67 clinical strains included 37 strains of AutB1 and 3 of AutB2, and the others were *autB*-null strains. The number of AAGC nucleotide repeats in our strains was 2–11 while in the former study was 3–19 (Arenas et al., 2016). With regard to the 207 carrier isolates in the former study, the number of the repeats was 2–22 and in most of the strains, the *autB* gene was disrupted by single-nucleotide insertions downstream of the repeats (Arenas et al., 2016). In the 111 isolates from carriers in our study, 25 isolates of CC4821 and 14 of UA were *autB*-null and isolated after 2005. The repeat number in the other 72 *autB*–positive isolates was also 2–11, where 60 harbored an AutB1 variant and the five isolates with 3 or 6 AAGC repeats were all disrupted by frame shift mutations. Meanwhile, another 12 AutB2 isolates belonged to CC174 (ST-6933) and UA. There were no AutB3 isolates in our strains (Table 1). Our study indicates that not all highly pathogenic strains are *autB*-positive and as expression is switched off in most *N. meningitidis* isolates, it can also be absent in some of the strains such as the CC4821 strains. Of 17 clinical strains of CC4821, 341215, and 440501, with the *autB* gene belonging to AutB1, two had repeat numbers of 8 and 4, respectively.

The sequences of the 66 *autB*-null strains between the *smpB* and *glcD* gene are all 968 bp long with no SNPs and these sequences are homologous to *N. lactamica* 020-06 (Figure 1C). The *autB* gene has been recognized as a pseudogene because it is not expressed in many meningococcal or gonococcal strains with the sequence (Ait-Tahar et al., 2000). Authors suggested this is probably due to the accumulation of mutations on *autB*, which with no need for coping with selective pressure (Ait-Tahar et al., 2000). Conversely, the high variability of the AutB passenger domain may be a reflection of the antigenic variability response to immune pressure, so that an immune response to AutB can be avoided, and where expression of the *autB* gene is switched off in most isolates (Arenas et al., 2016).

As has been verified in Arenas' study, the majority of *N. meningitidis* isolates equipped with the *autB* gene do not express AutB. One possible explanation is that some functions of AutB can be substituted by other ATs such as NalP, which cleaves the heparin-binding antigen NhbA or the α-peptide of IgA protease from the cell surface (Serruto et al., 2010; Roussel-Jazede et al., 2014). AutB is likely to be a backup mechanism when the concentration of these two proteins is low (Arenas et al., 2016). Nevertheless, the proportion of strains with the *autB* gene to *autB-*null strains is 24/27 (1956-2000), 20/31 (2001-2005), 49/85 (2006-2010), and 19/35 (2011-2015) in our study. In the 66 *autB-*null strains, only two have been isolated in 1985, one in 1988 and the others were all isolated after 2004. There are two hypothesis for this. First of this, it seems that the strains in China habitually lose the *autB* gene and the *autB* gene will not be retained as an intact gene in *N. meningitidis*. However, the number of strains in our study is relatively small so this hypothesis may be premature. Secondly, CC4821 strains are similar to *N. lactamica*, also shown in Figure 1A. In here, we have demonstrate that this is indeed a conservative feature of this CC, as we found in many isolates of this CC. Thus, in a common ancestor of this CC in China, the *autB* was lost by acquisition of *N. lactamica* sequences. Interestingly, two isolates of this CC seems to acquire *autB*, probably from another circulating strain as they harbor a typical and common AutB1 (see Table 1). This also supported by the similarity in the flanking regions with other CCs found in China and the predominance of this CC. Some isolates of UA, but no other CCs, do not have the *autB* gene but this is restricted uniquely to isolates of the same ST. Possibly, those isolates acquired sequences from the predominant clones of CC4821, which rarely have an *autB* gene.

It has been suggested that the *autB* gene in *Neisseria* was obtained from *Haemophilus* through domain shuffling and it has been proposed that the function of the 9 bp inverted repeat sequences of hUS at the beginning of the partial *holB* gene and the end of the partial *tmk* gene is recognition sites that enhance the transformation (Davis et al., 2001; Duffin and Seifert, 2010). In *autB*-null *N. meningitidis* and *N. lactamica* 020-06, the 10 or 12 bp DUS was located in the intergenic sequence (Figure 1C), according with the former study that most of the DUS (65%) in *Neisseria* is located in the intergenic sequences (Ambur et al., 2007). It has been postulated that the DUS may function to competitively inhibit transformation and this may be the mechanism of *autB*-null in these strains while strains without the DUS could gain the *autB* gene from other strains (Goodman and Scocca, 1988; Duffin and Seifert, 2010; Obergfell and Seifert, 2015).

In summary, we demonstrated the characterization and distribution of the *autB* gene and the variability of AutB in *N. meningitidis* isolates in China. Not all *N. meningitidis* strains were *autB*-positive in China. This study offers a new perspective on the *autB* gene and how it is possible that CC4821 and some of the UA *N. meningitidis* genomes could lose *autB* genes or acquire the alternative sequence from *N. lactamica*, the DUS may promote this process to some extent.

## Author contributions

ZS, BZ, and AZ designed the experiments; AZ, FS, LX, YG, and NX carried out the experiments; AZ, PZ, BZ, and ZS analyzed the experimental results; AZ wrote the manuscript; PZ, BZ, and ZS modified the manuscript. All authors have read and approved the manuscript.

### Conflict of interest statement

The authors declare that the research was conducted in the absence of any commercial or financial relationships that could be construed as a potential conflict of interest. The reviewer JC declared a shared affiliation, with no collaboration, with the authors to the handling Editor.
