# Supplementary material for: Characterization and Distribution of the autB Gene in Neisseria meningitidis
Source: Front Cell Infect Microbiol. 2017 Oct 6;7:436. doi: 10.3389/fcimb.2017.00436 (PMC5635059; doi:10.3389/fcimb.2017.00436)
Supplement: Supplementary file 1 [file Table1.DOCX]

**Table S1. Detailed information for the 178 *N. meningitidis* strains in this study**

| **Strain** | **disease** | **year** | **serogroup** | **genogroup** | **ST^a^** | **CC^b^** | **autB^c^** | **Phase^d^** | **Rn^e^** | **Remarks^f^** |
| --- | --- | --- | --- | --- | --- | --- | --- | --- | --- | --- |
| 11059 | carrier | 2011 | B | B | ST-5819 | UA | + | Out | 7 | B1 |
| 22135 | carrier | 2011 | B | B | ST-9920 | UA | + | Out | 4 | B1 |
| 34173 | carrier | 2011 | B | B | ST-9477 | CC4821 | - |  |  |  |
| 34279 | carrier | 2011 | B | B | ST-5542 | UA | - |  |  |  |
| 100503 | carrier | 2005 | C | C | ST-4894 | UA | + | Out | 7 | B1 |
| 100514 | carrier | 2005 | C | C | ST-4832 | CC4821 | - |  |  |  |
| 100517 | carrier | 2005 | NG | B | ST-12772 | UA | - |  |  |  |
| 100530 | carrier | 2005 | B | B | ST-5614 | CC4821 | - |  |  |  |
| 100572 | carrier | 2005 | B | B | ST-5610 | CC4821 | - |  |  |  |
| 100578 | carrier | 2005 | B | B | ST-5666 | UA | + | Out | 4 | B1 |
| 100595 | carrier | 2005 | B | B | ST-5609 | UA | + | Out | 8 | B1 |
| 100597 | carrier | 2005 | NG | cnl | ST-2146 | CC198 | + | Out | 2 | B1 |
| 100601 | patient | 2006 | C | C | ST-5463 | CC4821 | - |  |  |  |
| 100603 | patient | 2006 | C | C | ST-4821 | CC4821 | - |  |  |  |
| 100703 | patient | 2007 | X | X | ST-5944 | CC103 | + | Out | 4 | B1 |
| 101101 | carrier | 2011 | NG | E | ST-12760 | UA | + | Out | 7 | B1 |
| 101108 | patient | 2009 | C | C | ST-2724 | CC11 | + | Out | 11 | B1 |
| 130508 | patient | 2005 | A | A | ST-7 | CC5 | + | Out | 4 | B1 |
| 130708 | carrier | 2007 | NG | C | ST-4821 | CC4821 | - |  |  |  |
| 130711 | carrier | 2007 | NG | B | ST-12274 | CC4821 | - |  |  |  |
| 130712 | carrier | 2007 | NG | cnl | ST-2146 | CC198 | + | Out | 2 | B1 |
| 130724 | carrier | 2007 | NG | C | ST-4821 | CC4821 | - |  |  |  |
| 130803 | carrier | 2008 | C | C | ST-6928 | CC4821 | - |  |  |  |
| 130807 | carrier | 2008 | NG | E | ST-8238 | UA | + | Out | 5 | B1 |
| 130808 | carrier | 2008 | NG | B | ST-5819 | UA | + | Out | 4 | B1 |
| 130811 | carrier | 2008 | NG | C | ST-5473 | CC4821 | - |  |  |  |
| 130814 | carrier | 2008 | NG | C | ST-5473 | CC4821 | - |  |  |  |
| 130817 | carrier | 2008 | NG | cnl | ST-2146 | CC198 | + | Out | 2 | B1 |
| 130903 | carrier | 2009 | NG | W | ST-8491 | CC4821 | - |  |  |  |
| 130907 | carrier | 2009 | NG | C | ST-4821 | CC4821 | - |  |  |  |
| 130927 | carrier | 2009 | NG | Y | ST-9756 | CC175 | + | In | 3 | Fs, B1 |
| 130930 | carrier | 2009 | NG | X | ST-12761 | UA | + | In | 6 | B1 |
| 130931 | carrier | 2009 | NG | X | ST-12761 | UA | + | Out | 7 | B1 |
| 130933 | carrier | 2009 | C | C | ST-175 | CC175 | + | Out | 5 | B1 |
| 130936 | carrier | 2009 | NG | C | ST-10755 | UA | - |  |  |  |
| 130943 | carrier | 2009 | NG | W | ST-8242 | CC4821 | - |  |  |  |
| 131123 | carrier | 2011 | C | C | ST-2493 | CC32 | + | Out | 7 | B1 |
| 131148 | carrier | 2011 | C | C | ST-9936 | CC4821 | - |  |  |  |
| 140507 | carrier | 2005 | NG | Y | ST-175 | CC175 | + | In | 3 | Fs, B1 |
| 140511 | carrier | 2005 | NG | B | ST-12779 | UA | + | Out | 4 | B2 |
| 140901 | carrier | 2009 | NG | B | ST-8241 | CC4821 | - |  |  |  |
| 141308 | carrier | 2013 | NG | B | ST-5819 | UA | + | Out | 7 | B1 |
| 141401 | carrier | 2013 | NG | B | ST-12782 | UA | + | In | 3 | Fs, B1 |
| 150703 | carrier | 2006 | NG | cnl | ST-2146 | CC198 | + | Out | 2 | B1 |
| 150717 | carrier | 2007 | NG | B | ST-6934 | UA | + | Out | 8 | B2 |
| 150801 | carrier | 2008 | NG | cnl | ST-8243 | CC198 | + | Out | 2 | B1 |
| 150808 | carrier | 2008 | NG | B | ST-5863 | UA | + | In | 3 | Fs, B2 |
| 210622 | carrier | 2006 | C | C | ST-5750 | UA | - |  |  |  |
| 210711 | carrier | 2007 | NG | B | ST-6934 | UA | + | Out | 8 | B2 |
| 210713 | carrier | 2007 | NG | C | ST-3436 | CC4821 | - |  |  |  |
| 210902 | patient | 2008 | B | B | ST-8919 | UA | - |  |  |  |
| 211004 | carrier | 2009 | NG | cnl | ST-2146 | CC198 | + | Out | 2 | B1 |
| 211110 | carrier | 2010 | NG | E | ST-12773 | UA | + | Out | 5 | B1 |
| 211351 | carrier | 2013 | NG | X | ST-5604 | UA | + | Out | 7 | B1 |
| 220601 | patient | 2006 | C | C | ST-4821 | CC4821 | - |  |  |  |
| 220808 | carrier | 2007 | NG | E | ST-12763 | UA | + | Out | 7 | B1 |
| 220814 | carrier | 2007 | NG | Y | ST-12774 | CC92 | + | Out | 4 | B1 |
| 221504 | carrier | 2015 | NG | B | ST-12764 | UA | + | Out | 7 | B1 |
| 231011 | carrier | 2009 | NG | B | ST-12301 | UA | + | Out | 5 | B1 |
| 310501 | patient | 2005 | A | A | ST-7 | CC5 | + | Out | 4 | B1 |
| 310502 | patient | 2005 | C | C | ST-658 | CC11 | + | Out | 5 | B1 |
| 310803 | patient | 2006 | B | B | ST-5751 | UA | - |  |  |  |
| 310812 | patient | 2006 | B | B | ST-6930 | CC32 | + | Out | 7 | B1 |
| 310819 | carrier | 2007 | NG | C | ST-4821 | CC4821 | - |  |  |  |
| 310822 | patient | 2007 | B | B | ST-8815 | UA | - |  |  |  |
| 311105 | carrier | 2010 | NG | C | ST-9589 | UA | + | Out | 7 | B1 |
| 311112 | patient | 2011 | B | B | ST-5798 | CC4821 | - |  |  |  |
| 311113 | patient | 2011 | B | B | ST-9454 | CC4821 | - |  |  |  |
| 311114 | patient | 2011 | B | B | ST-9455 | CC4821 | - |  |  |  |
| 320501 | patient | 2005 | C | C | ST-4820 | CC4821 | - |  |  |  |
| 320503 | patient | 2005 | C | C | ST-4821 | CC4821 | - |  |  |  |
| 320524 | patient | 2005 | A | A | ST-5084 | CC5 | + | Out | 4 | B1 |
| 320802 | carrier | 2008 | NG | C | ST-4821 | CC4821 | - |  |  |  |
| 320805 | carrier | 2008 | NG | Y | ST-175 | CC175 | + | In | 3 | Fs, B1 |
| 320807 | carrier | 2008 | NG | B | ST-12765 | UA | - |  |  |  |
| 321109 | carrier | 2011 | NG | B | ST-5662 | UA | - |  |  |  |
| 321112 | carrier | 2011 | NG | C | ST-12303 | UA | + | Out | 7 | B2 |
| 321114 | patient | 2011 | B | B | ST-3200 | CC4821 | - |  |  |  |
| 330505 | patient | 2005 | C | C | ST-4896 | CC4821 | - |  |  |  |
| 330705 | carrier | 2006 | NG | cnl | ST-2146 | CC198 | + | Out | 2 | B1 |
| 330742 | carrier | 2007 | A | A | ST-7 | CC5 | + | Out | 4 | B1 |
| 340542 | patient | 2004 | C | C | ST-4821 | CC4821 | - |  |  |  |
| 340552 | carrier | 2005 | B | B | ST-4897 | CC4821 | - |  |  |  |
| 340562 | patient | 2005 | A | A | ST-5083 | CC5 | + | Out | 4 | B1 |
| 341215 | patient | 2012 | B | B | ST-4821 | CC4821 | + | Out | 8 | B1 |
| 350602 | patient | 2006 | C | C | ST-5751 | UA | - |  |  |  |
| 350703 | patient | 2007 | B | B | ST-8789 | UA | - |  |  |  |
| 360522 | carrier | 2005 | NG | C | ST-4821 | CC4821 | - |  |  |  |
| 360527 | carrier | 2005 | B | B | ST-5113 | UA | + | Out | 4 | B1 |
| 360559 | carrier | 2005 | B | B | ST-5617 | UA | + | Out | 7 | B1 |
| 360564 | carrier | 2005 | NG | B | ST-5542 | UA | - |  |  |  |
| 360601 | patient | 2006 | C | C | ST-5540 | UA | + | Out | 5 | B1 |
| 360624 | carrier | 2006 | C | C | ST-5473 | CC4821 | - |  |  |  |
| 370537 | carrier | 2005 | B | B | ST-5611 | UA | + | Out | 5 | B1 |
| 370601 | patient | 2006 | C | C | ST-3200 | CC4821 | - |  |  |  |
| 371104 | carrier | 2011 | NG | E | ST-5586 | UA | + | Out | 7 | B1 |
| 371401 | patient | 2014 | W | W | ST-11 | CC11 | + | Out | 8 | B1 |
| 371402 | patient | 2014 | W | W | ST-11 | CC11 | + | Out | 8 | B1 |
| 371404 | carrier | 2014 | W | W | ST-11 | CC11 | + | Out | 8 | B1 |
| 371428 | carrier | 2014 | W | W | ST-11 | CC11 | + | Out | 8 | B1 |
| 420601 | carrier | 2006 | B | B | ST-5567 | UA | + | Out | 5 | B1 |
| 420707 | patient | 2006 | B | B | ST-5615 | UA | - |  |  |  |
| 420710 | patient | 2007 | B | B | ST-8791 | UA | + | Out | 8 | B1 |
| 420713 | carrier | 2007 | NG | E | ST-5586 | UA | + | Out | 7 | B1 |
| 420718 | patient | 2007 | C | C | ST-4821 | CC4821 | - |  |  |  |
| 420815 | carrier | 2008 | X | X | ST-5586 | UA | + | Out | 7 | B1 |
| 420818 | carrier | 2008 | E | E | ST-5586 | UA | + | Out | 7 | B1 |
| 421006 | carrier | 2010 | NG | B | ST-12766 | UA | - |  |  |  |
| 421007 | carrier | 2010 | NG | B | ST-4821 | CC4821 | - |  |  |  |
| 421014 | patient | 2010 | B | B | ST-6934 | UA | + | Out | 7 | B2 |
| 421103 | carrier | 2011 | NG | B | ST-10746 | UA | + | Out | 4 | B2 |
| 421106 | carrier | 2011 | NG | C | ST-5542 | UA | - |  |  |  |
| 421202 | carrier | 2012 | NG | E | ST-5586 | UA | + | Out | 7 | B1 |
| 421205 | carrier | 2012 | NG | B | ST-12776 | UA | + | Out | 5 | B1 |
| 431401 | carrier | 2014 | NG | C | ST-4821 | CC4821 | - |  |  |  |
| 431410 | carrier | 2014 | NG | C | ST-4821 | CC4821 | - |  |  |  |
| 431419 | carrier | 2014 | NG | C | ST-5542 | UA | - |  |  |  |
| 431421 | carrier | 2014 | NG | B | ST-12767 | CC4821 | - |  |  |  |
| 431423 | carrier | 2014 | NG | Y | ST-7156 | CC175 | + | In | 3 | Fs, B1 |
| 440501 | patient | 2005 | C | C | ST-4831 | CC4821 | + | Out | 4 | B1 |
| 440529 | patient | 2005 | A | A | ST-7 | CC5 | + | Out | 4 | B1 |
| 440530 | patient | 2005 | A | A | ST-7 | CC5 | + | Out | 4 | B1 |
| 440550 | carrier | 2005 | C | C | ST-5587 | UA | + | Out | 5 | B1 |
| 440716 | patient | 2006 | B | B | ST-3789 | UA | + | Out | 7 | B1 |
| 440725 | carrier | 2006 | B | B | ST-5620 | UA | + | Out | 4 | B1 |
| 440902 | patient | 2008 | B | B | ST-4821 | CC4821 | - |  |  |  |
| 440910 | patient | 2009 | B | B | ST-8918 | CC41/44 | + | Out | 7 | B2 |
| 440911 | patient | 2009 | B | B | ST-5662 | UA | - |  |  |  |
| 441005 | patient | 2010 | B | B | ST-5662 | UA | - |  |  |  |
| 441009 | patient | 2010 | B | B | ST-8667 | UA | + | Out | 4 | B1 |
| 441102 | patient | 2010 | B | B | ST-8929 | UA | + | Out | 7 | B1 |
| 441107 | patient | 2011 | B | B | ST-9456 | CC4821 | - |  |  |  |
| 441207 | patient | 2011 | B | B | ST-9921 | UA | - |  |  |  |
| 510510 | carrier | 2005 | B | B | ST-5635 | CC41/44 | + | Out | 4 | B1 |
| 510608 | carrier | 2006 | B | B | ST-32 | CC32 | + | Out | 7 | B1 |
| 510612 | patient | 2006 | A | A | ST-7 | CC5 | + | Out | 4 | B1 |
| 520701 | carrier | 2006 | NG | B | ST-5740 | UA | + | Out | 5 | B1 |
| 520814 | carrier | 2007 | NG | cnl | ST-2146 | CC198 | + | Out | 2 | B1 |
| 520822 | carrier | 2007 | NG | W | ST-6933 | CC174 | + | Out | 11 | B2 |
| 520826 | carrier | 2007 | NG | W | ST-6933 | CC174 | + | Out | 11 | B2 |
| 520837 | carrier | 2007 | NG | W | ST-6933 | CC174 | + | Out | 11 | B2 |
| 530605 | carrier | 2006 | B | B | ST-5568 | UA | - |  |  |  |
| 640704 | carrier | 2007 | NG | B | ST-5615 | UA | - |  |  |  |
| 640705 | carrier | 2007 | NG | C | ST-3256 | UA | + | Out | 5 | B1 |
| 641201 | carrier | 2008 | NG | C | ST-12305 | UA | + | Out | 7 | B2 |
| 11300180 | carrier | 2009 | B | B | ST-8674 | CC41/44 | + | Out | 4 | B1 |
| 13200133 | carrier | 2009 | B | B | ST-5636 | UA | + | Out | 4 | B1 |
| 13200163 | carrier | 2009 | B | B | ST-8689 | UA | - |  |  |  |
| GDL-MG672 | patient | 1984 | A | A | ST-3 | CC1 | + | Out | 4 | B1 |
| GX2002-2 | patient | 2002 | A | A | ST-7 | CC5 | + | Out | 4 | B1 |
| GX526 | patient | 1982 | A | A | ST-3 | CC1 | + | Out | 4 | B1 |
| GZ80003 | patient | 1956 | A | A | ST-5 | CC5 | + | Out | 4 | B1 |
| GZ80025 | patient | 1974 | A | A | ST-5239 | CC1 | + | Out | 4 | B1 |
| GZ80026 | patient | 1974 | A | A | ST-5240 | CC1 | + | Out | 4 | B1 |
| GZ80028 | patient | 1974 | A | A | ST-5243 | CC1 | + | Out | 4 | B1 |
| GZ80056 | carrier | 1963 | A | A | ST-5079 | CC5 | + | Out | 4 | B1 |
| HA80132 | carrier | 1980 | B | B | ST-5630 | UA | + | Out | 8 | B1 |
| HA84024 | patient | 1984 | A | A | ST-5251 | CC5 | + | Out | 4 | B1 |
| HB79049 | patient | 1979 | B | B | ST-230 | CC32 | + | Out | 10 | B1 |
| HE84005 | patient | 1984 | A | A | ST-203 | CC5 | + | Out | 4 | B1 |
| HE88001 | patient | 1988 | B | B | ST-3482 | UA | - |  |  |  |
| HE89009 | patient | 1989 | B | B | ST-5642 | UA | + | Out | 7 | B1 |
| JX213099 | patient | 1980 | A | A | ST-5249 | CC1 | + | Out | 4 | B1 |
| JX80281 | carrier | 1980 | B | B | ST-5668 | UA | + | Out | 4 | B2 |
| LN16 | carrier | 1986 | B | B | ST-5248 | UA | + | Out | 5 | B1 |
| LN9233 | carrier | 1992 | B | B | ST-658 | CC11 | + | Out | 5 | B1 |
| LN95 | carrier | 1986 | B | B | ST-5670 | UA | + | Out | 4 | B2 |
| NX08 | patient | 1984 | B | B | ST-5601 | UA | + | Out | 4 | B2 |
| NX17 | patient | 1985 | B | B | ST-5657 | CC269 | + | Out | 5 | B1 |
| NX21 | patient | 1987 | B | B | ST-5655 | CC8 | + | Out | 5 | B1 |
| SC84082 | carrier | 1984 | B | B | ST-11 | CC11 | + | Out | 10 | B1 |
| SH1985 | patient | 1985 | B | B | ST-3129 | UA | - |  |  |  |
| SH261 | patient | 1977 | B | B | ST-44 | CC41/44 | + | Out | 5 | B1 |
| SH80090 | patient | 1979 | A | A | ST-5242 | CC1 | + | In | 3 | B1 |
| SHG255 | patient | 1985 | B | B | ST-5658 | UA | - |  |  |  |
| SX09162 | carrier | 2009 | B | B | ST-4690 | UA | + | Out | 5 | B1 |
| sy03 | carrier | 2012 | B | B | ST-5662 | UA | - |  |  |  |
| XJ043 | patient | 1986 | A | A | ST-3 | CC1 | + | Out | 4 | B1 |

^a^ ST, sequence type.

^b^ CC, clonal complex.

^c^ +, *autB* positive; -, *autB* null.

^d^ In/Out of frame was considered according to the number of AAGC repeats.

^e^ Rn, number of AAGC repeats.

^f^ Other genetic features. The passenger domains of AutB proteins are clustered in three variants indicated as B1, B2 and B3. Fs, frameshifts.
